# Overexpression of the Synthetic Chimeric Native-T-phylloplanin-GFP Genes Optimized for Monocot and Dicot Plants Renders Enhanced Resistance to Blue Mold Disease in Tobacco (*N. tabacum* L.)

**DOI:** 10.1155/2014/601314

**Published:** 2014-03-20

**Authors:** Dipak K. Sahoo, Sumita Raha, James T. Hall, Indu B. Maiti

**Affiliations:** ^1^Kentucky Tobacco Research and Development Center, College of Agriculture, Food and Environment, University of Kentucky, Lexington, KY 40546-0236, USA; ^2^Department of Radiation Oncology, Feinberg School of Medicine, Northwestern University, Ward-13-002, 303 East Chicago Avenue, Chicago, IL 60611, USA

## Abstract

To enhance the natural plant resistance and to evaluate the antimicrobial properties of phylloplanin against blue mold, we have expressed a synthetic chimeric native-phylloplanin-GFP protein fusion in transgenic *Nicotiana tabacum* cv. KY14, a cultivar that is highly susceptible to infection by *Peronospora tabacina*. The coding sequence of the tobacco phylloplanin gene along with its native signal peptide was fused with GFP at the carboxy terminus. The synthetic chimeric gene (native-phylloplanin-GFP) was placed between the modified *Mirabilis mosaic virus* full-length transcript promoter with duplicated enhancer domains and the terminator sequence from the rbcSE9 gene. The chimeric gene, expressed in transgenic tobacco, was stably inherited in successive plant generations as shown by molecular characterization, GFP quantification, and confocal fluorescent microscopy. Transgenic plants were morphologically similar to wild-type plants and showed no deleterious effects due to transgene expression. Blue mold-sensitivity assays of tobacco lines were performed by applying *P. tabacina* sporangia to the upper leaf surface. Transgenic lines expressing the fused synthetic native-phyllopanin-GFP gene in the leaf apoplast showed resistance to infection. Our results demonstrate that *in vivo* expression of a synthetic fused native-phylloplanin-GFP gene in plants can potentially achieve natural protection against microbial plant pathogens, including *P. tabacina* in tobacco.

## 1. Introduction

Downy mildew disease of cultivated tobacco (*Nicotiana tabacum* L.), commonly known as blue mold, is caused by the obligately biotrophic oomycete pathogen* Peronospora tabacina *D.B. Adam. Blue mold was first reported in tobacco-growing areas around the end of the 19th century in Australia and Argentina [1]. In 1921, it was first seen in tobacco seedbeds in the United States in the state of Georgia. In 1979, blue mold epidemics resulted in annual crop losses exceeding $250 million in the eastern United States and Canada [1]. During periods of cool and wet weather,* P. tabacina *can complete its lifecycle in less than 10 days, and the disease becomes polycyclic, resulting in a continuous production of infective asexual sporangia (up to 10^6^/cm^2^ of infected leaf tissue), which can cause widespread blue mold epidemics [1].

Pathogenic fungi and fungal-like organisms cause about 20% of annual crop losses worldwide [3]. To control plant diseases, large amounts of chemically synthesized fungicides are used at present in agriculture in both developed and developing countries. Chemical treatment using the systemic fungicide metalaxyl can effectively control blue mold disease on tobacco; however, the long-term use of chemical pesticides can result in the development of resistance in pathogens and can also have an adverse impact on human health and the environment. It has been reported that North and Central American isolates of* P. tabacina* have developed resistance to metalaxyl [4]. Such agricultural practice is causing environmental pollution and animal/human diseases and hazardous effects. To avoid or minimize the use of chemically synthesized fungicides, we need to develop alternative fungicides suitable for the environment and human health. Hence, the employment of host plant resistance is the most economic and environmentally sustainable means for controlling blue mold.

Several* Nicotiana* species of Australian origin, such as* N. debneyi*,* N. exigua*,* N. goodspeedii*,* N. maritime*,* N. megalosiphon*,* N. rotundifolia,* a noncultivated tobacco species* N. megalosiphon* from Cuba, and the wild species* N. langsdorffii* from South America possess genetic resistance to blue mold [1, 5]. Although host resistance to* P. tabacina *infection is low in* N. tabacum*, the species still possesses several defense responses against* P. tabacina *[6, 7]. Identification and isolation of resistance gene(s) from these species will provide valuable tools for developing transgene-mediated cultivars of tobacco that can resist blue mold infection.

Different types of plant proteins possessing antifungal properties have been identified and studied extensively in the past two decades. Examples are chitinases and chitinase-like proteins with antifungal activity towards* Fusarium oxysporum* and* Rhizoctonia solani* [8, 9], cyclophilin-like proteins with antifungal activities [10, 11], defensins and defensin-like peptides that inhibit growth of* F*.* oxysporum* and* M*.* arachidicola* [12],* Asparagus *deoxyribonuclease that exhibits antifungal activity against* Botrytis cinerea* [13], ginkbilobin that has strong antifungal action against* B. cinerea*,* Coprinus comatus*,* F*.* oxysporum*, and* R*.* solani* [14], and glucanases that are active against* Alternaria longipes*,* Rhizoctonia cerealis*,* V. dahlia*, and* Fusarium oxysporum* [15]. Topical application of certain antifungal peptides/proteins on plants appears to provide a first-line-of-defense/resistance towards a number of pathogenic fungi and fungal-like organisms. Plants producing such antifungal peptides/proteins in surface tissues (intracellular or intercellular spaces or the apoplast) might provide endogenous resistance or tolerance to invading fungi.

About 30% of vascular plants possess glandular secreting trichomes; these can include tall glandular secreting trichomes (TGSTs) and short glandular trichomes (SGTs). Glandular head cells of TGSTs have been shown to synthesize diterpenoids and sugar esters [16]. In tobacco, short glandular trichomes (SGTs) synthesize unique proteins known as T-phylloplanins [17, 18]. T-phylloplanin proteins secreted onto the surfaces of tobacco leaves have antimicrobial properties and have been shown to inhibit blue mold disease caused by* P. tabacina *[17–19]. Application of tobacco phylloplanin to turfgrass also inhibits gray leaf and brown patch diseases caused by the ascomycete* Pyricularia oryzae* and the basidiomycete* Rhizoctonia solani* [20]. Recently, it has been demonstrated that the mature tobacco phylloplanin gene without its own signal peptide fused with GFP and targeted to the apoplasm increases resistance to blue mold disease in tobacco [21].

In the present study, we generated chimeric gene constructs of synthetic native T-phylloplanin fused to GFP (nat-T-phyllo-GFP) to differentiate from the endogenous phylloplanin gene products. The codons of the native T-phylloplanin-GFP gene fusion constructs were optimized separately for dicot and monocot plants. This synthetic gene has minimal sequence homology with the endogenous gene and is expected to be less susceptible to posttranscriptional gene silencing* in vivo*. We report here the overexpression of the synthetic native T-phylloplanin (with its native signal peptide) fused GFP gene in tobacco cultivar KY14. Transgenic lines expressing the fused synthetic chimeric nat-T-phyllo-GFP gene showed resistance against blue mold infection.

## 2. Materials and Methods

### 2.1. Chemicals and Enzymes

All chemicals and reagents used were of analytical grade or higher and were obtained from Sigma-Aldrich, Fisher Scientific, and BDH, as applicable. DNA modifying enzymes and restriction enzymes were purchased from Invitrogen Life Technologies (USA). Nitrocellulose membranes for western blot analysis were obtained from Schleicher & Schuell (Keene, NH, USA).

### 2.2. Construction of Plant Expression Vectors pKM24-ibm8 and pKM24-ibm10

**Figure 1:**
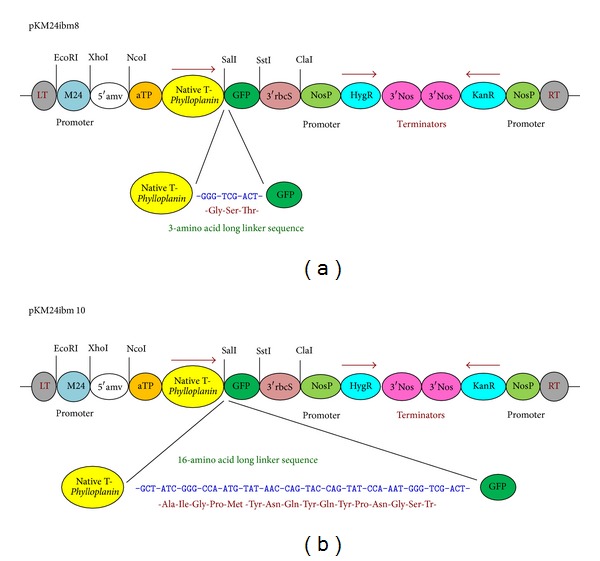
Schematic map of the plant expression vector constructs pKM24-ibm8 and pKM24-ibm10 containing the synthetic tobacco* Phylloplanin *gene (GenBank accession no. AY705384) fused in-frame with GFP. Two genes (native T-phylloplanin and GFP) were fused in-frame in constructs pKM24-ibm8 and pKM24-ibm10 with linkers of 3 and 16 amino acids, respectively. The modified full-length transcript promoter (M24) of* Mirabilis mosaic virus* [2] directs the coding sequences of the respective native phylloplanin-GFP gene fusions. The chimeric gene sequence native T-phylloplanin-GFP was codon-optimized for both the dicot tobacco (pKM24ibm8; GenBank accession KF951257) and the monocot bent grass (pKM24ibm10; GenBank accession KF951258). A translational enhancer sequence (5′amv), the 35-nt long 5′-untranslated region of AlMV RNA 4, was fused with the gene. The apoplast targeting sequence (aTP) of the* Arabidopsis* 2S2 protein gene was fused in-frame with the coding sequence of native T-phylloplanin fused with GFP in the constructs. LT, left T-DNA border; RT, right T-DNA border; KanR, neomycin phosphotransferase II marker gene, and hygromycin resistance (HgR) directed by the nopaline synthase promoter (NosP), the 3′-terminator sequences (terminators) of the ribulose bisphosphate carboxylase small subunit (3′RbcS) and nopaline synthase (3′Nos) genes are also shown. The EcoRI, XhoI, SstI, NcoI, and ClaI restriction sites used to assemble these expression vectors are shown.

The chimeric gene constructs were designed using the tobacco native phylloplanin gene (GenBank accession no. AY705384) fused with GFP; codon choices were optimized for the dicot species tobacco (*Nicotiana tabacum*) and the monocot species creeping bentgrass (*Agrostis stolonifera*). A translational enhancer sequence (5′amv), the 35-nt long 5′-untranslated region of AlMV RNA 4, was fused with the chimeric phylloplanin gene. The apoplast targeting sequence (aTP) of the Arabidopsis 2S2 protein gene was fused with the coding sequence of phylloplanin containing its native signal peptide (SP) fused with GFP [22]. The fused synthetic chimeric native T-phylloplanin-GFP (nat-T-phyllo-GFP) genes were synthesized by GeneArt (Invitrogen, Life technologies, USA, http://www.lifetechnologies.com/GeneArt/). Each modified synthetic nat-T-phyllo-GFP gene fragment that was codon optimized for either monocots or dicots was cloned separately into the XhoI/SstI sites of Bluescript (KS+) to generate plasmids pBibm8 and pBibm10, respectively. Before use, the sequences of both fragments were confirmed. The 5′-Xho1-SstI-3′ fragments were gel purified and cloned into the corresponding sites in the binary vector pKM24KH (GenBank accession HM036220) to generate the plasmids pKM24-ibm8 (GenBank accession KF951257) and pKM24-ibm10 (GenBank accession KF951258). The resulting plasmids have the following general structure: 5′-EcoR1-M24-promoter-HindIII-XhoI-5′amv-aTP-SP-phyllo-GFP-SstI-3′ (Figure 1). The modified full-length transcript promoter (M24) of the* Mirabilis mosaic virus* [2, 21, 23, 24] directs expression of the coding sequences of the nat-T-phyllo-GFP gene fusions.

### 2.3. Plant Material and Agrobacterium-Mediated Plant Transformation

A burley tobacco line (*Nicotiana tabacum* cv. KY14) that is highly sensitive to* P. tabacina* infection was used for plant transformation. Each of the two constructs, pKM24-ibm8 and pKM24-ibm10, was introduced into the* Agrobacterium tumefaciens* strain C58C1 : pGV3850 by the freeze thaw method [25], and* Agrobacterium tumefaciens*-mediated tobacco transformation was performed as described previously [26]. Ten independent plant lines (R_0_ lines, 1st generation progeny) were generated for each construct. Regenerated kanamycin-resistant plants were grown in the greenhouse [27]; seeds were collected from self-pollinated primary transformants. Transgenic tobacco seeds (R_1_) were germinated in the presence of kanamycin (300 mg/L). Transgenic lines (R_1_ progeny, 2nd generation) with KanR/KanS segregation ratios of 3 : 1 were selected for further analysis.

### 2.4. Analysis of Transgenic Plants

#### 2.4.1. Polymerase Chain Reaction (PCR)

Integration and transcription of the fused nat-T-phyllo-GFP constructs pKM24-ibm8 and pKM24-ibm10 in transgenic plants (T_1_ and T_2_) were analyzed by PCR, RT-PCR, and real-time qRT-PCR assays using appropriately-designed gene-specific primers (Table 1). Genomic DNA from untransformed control plants, control plants transformed with the empty vector, and the pKM24-ibm8 and pKM24-ibm10 transformants was isolated using a DNeasy Plant Mini Kit (Qiagen, Valencia, CA) as described earlier [23].

#### 2.4.2. RNA Isolation, Real-Time RT-PCR

Total cellular RNA from transgenic tobacco seedlings generated with the constructs pKM24-ibm8 and pKM24-ibm10 was isolated using the RNeasy Plant Mini kit (Qiagen, Chatsworth, USA) as described in [24]. Total RNA (2 *μ*g samples) was treated with RNase-free DNase (Sigma, USA) per the manufacturer's instructions and was used for synthesis of first-strand cDNA with the iScript cDNA synthesis kit (Bio-Rad, USA) in a total volume of 20 *μ*L following the manufacturer's instructions. For the no-reverse-transcriptase control, an individual reaction was performed in parallel without the addition of reverse transcriptase. One twentieth (1 *μ*L) of the RT reaction was used in the subsequent PCR reaction with gene-specific primers for nat-T-phyllo-GFP (#1 and #2) to detect the nat-T-phyllo-GFP-specific mRNA. As a negative control, each primer pair was tested against DNase-treated RNA to confirm cDNA dependence on the amplification. PCR products were examined on an ethidium bromide-stained agarose gel.

#### 2.4.3. Quantitative Real-Time Polymerase Chain Reaction (qRT-PCR)

The qRT-PCR reactions were performed in three biological replicates using total RNA samples extracted from three independent plants grown under identical conditions. The expression level of nat-T-phyllo-GFP mRNA in transgenic plants was evaluated by real-time quantitative RT-PCR. For qRT-PCR, gene-specific primers for native T-phylloplanin (#1 and #3) were used to evaluate T-phyllo-GFP transcript levels. The qRT-PCR assays were performed using iTaq SYBR Green Supermix with ROX (Bio-Rad, USA) according to the manufacturer's instructions. The tobacco tubulin gene (primers #4 and #5) was used as an internal control to normalize the expression of T-phyllo-GFP. The comparative Ct threshold cycle method (Applied Biosystems bulletin) was used to evaluate the relative expression levels of the transcripts. The threshold cycle was automatically determined for each reaction using default parameters (Step One Real-Time PCR System, Applied Biosystems). The PCR specificity was determined by melt curve analysis of the amplified products using the standard method installed in the System (Step One Real-Time PCR System, Applied Biosystems).

### 2.5. Extracellular Fluid Extraction from Leaves

Extracellular fluid (EF) was extracted from leaves of transgenic and control plants grown in the greenhouse for 8 weeks as described earlier [21]. Total soluble leaf extract (TP), extracellular fluid extract (EF), and soluble postextracellular fluid (PEF) extracts were obtained from leaves of transgenic and control plants as described earlier [21] to estimate the amount of expressed GFP present.

### 2.6. Green Fluorescent Protein (GFP) Assay

Eight-week-old transgenic and control plants grown in the greenhouse were sampled for protein extraction. To obtain total soluble leaf extract (TP) and soluble postextracellular fluid extract (PEF), the leaf was homogenized in 5 mM Hepes/NaOH, pH 6.3, containing 50 mM NaCl [21, 28] before and after collection of extracellular fluid, respectively, and centrifuged at 10,000 ×g for 20 min at 4°C to collect the soluble fraction. The protein contents of the plant extracts were determined according to the Bradford method using BSA as the standard [29]. TP, EF, and PEF samples were further diluted with 0.1 M Na_2_CO_3_, pH 9.6, to estimate GFP concentrations [30].

Fluorometric quantification of GFP was done by following the method described earlier [30]. The GFP concentration (expressed in *μ*g GFP per mg protein) was measured in leaf protein extracts from transgenic and control plants with the Turner Biosystems Luminometer using the GFP-UV module. The results were expressed as means ± standard deviation of readings from ten different plants of the same line (three readings were taken per plant).

### 2.7. Western Blot Analysis

Crude extracts of control and transgenic tobacco plants were prepared as described earlier [23, 24]. Whole seedlings were homogenized in a mortar and pestle at 4°C in a protein extraction buffer (0.3 M NaCl, 0.1 M Tris-HCl pH 8.0, 5 mM PMSF, 10 *μ*g/mL each benzamidine, trypsin inhibitor, bacitracin, leupeptin, pepstatin A). Extracts were centrifuged at 10,000 ×g for 15 min at 4°C to obtain crude extract supernatants. Protein contents were determined by the Bradford method using BSA as standard [29]. Proteins (50 *μ*g per lane) were separated by SDS-polyacrylamide gel electrophoresis [31], transferred to a nitrocellulose membrane (Bio-Rad) and subjected to Western blot analysis as described earlier [23]. For nat-T-phyllo-GFP detection, the membrane was incubated with primary anti-GFP polyclonal antibody (1 : 5000), then with horseradish peroxidase-conjugated anti-rabbit secondary antibody (1 : 5000), and was developed by using a chemiluminescent reagent (Pierce, Supersignal West Pico Chemiluminescent substrate).

### 2.8. GFP Visualization by Confocal Laser Scanning Microscopy

The fluorescence images of transgenic tobacco expressing nat-T-phyllo-GFP and control tobacco plants were captured with confocal laser scanning microscope (TCS SP5; Leica Microsystems CMS GmbH, D-68165 Mannheim, Germany) using LAS AF (Leica Application Suite Advanced Fluorescence) 1.8.1 build 1390 software. We used the PL FLUOTAR objective (10.0X/N.A.0.3 DRY) with confocal pinhole set at Airy 1 and 1x zoom factor for improved resolution with eight bits. GFP expressed in transgenic plants was excited with an argon laser (30%) with AOTF for 488 nm (at 40%) [32], and the fluorescence emissions were collected between 501 and 580 nm with the photomultiplier tube (PMT) detector gain set at 1150V.

### 2.9. Plant Inoculations and* P. tabacina *Infection Assays

The* P. tabacina *isolate KY 79 was used in this study. Isolate KY 79 was originally collected from a tobacco field near Georgetown, KY, in 1979. The pathogen was maintained by weekly serial passage on* N. tabacum* cv. KY 14 plants (7–12-week-old) as described earlier [33]. A water suspension containing fresh sporangiospores of KY79 (10^5^ sporangiospores/mL) was used for challenge inoculations by drop inoculation as described earlier [1]. In brief, leaves of control and transgenic* N. tabacum* cv. KY 14 plants (six to seven weeks old) were inoculated by applying a 3 *μ*L drop of the sporangial suspension directly onto the adaxial surface of the leaf panels (8–10 sites/leaf, three leaves/plant). Inoculated plants were placed in sealed premoistened plastic tubs in the dark and kept overnight before being transferred to a growth chamber specifically designed for blue mold containment. One week after inoculation, plant reaction to blue mold infection was evaluated.

Any leaves that showed signs of blue mold infection were collected and incubated overnight in a humid chamber in the dark to induce sporulation. Zones of sporulation were measured and the spore concentrations per leaf were calculated for both control and transgenic plants.

## 3. Results

### 3.1. Molecular Analysis of Transgenic Plants Carrying the Fused Synthetic Chimeric T-phylloplanin-GFP Gene

**Figure 2:**
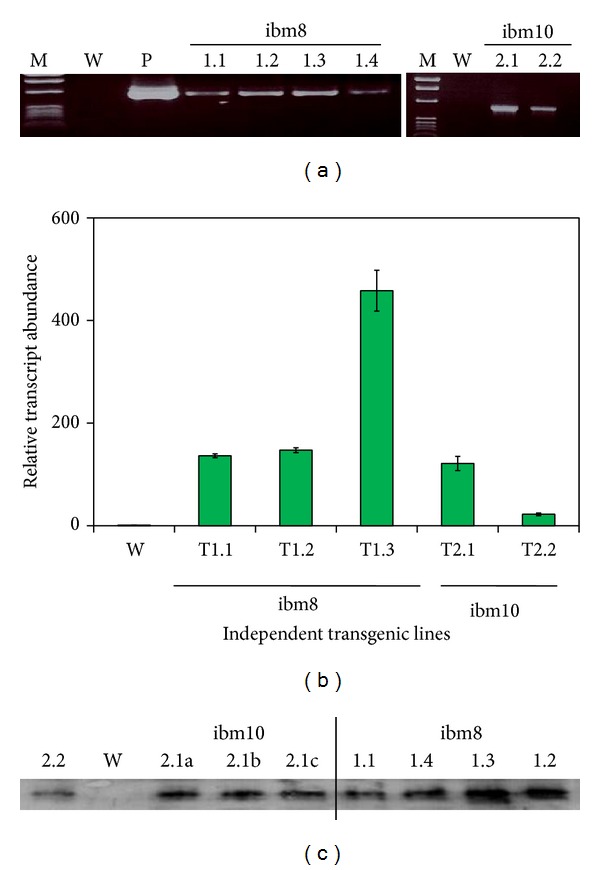
Expression analyses of transgenic plants containing the chimeric nat-T-phylloplanin-GFP gene. (a) Gene integration and expression analysis was determined by reverse transcriptase-polymerase chain reaction (RT-PCR); amplification products from independent transgenic lines (R_1_ progeny, 2nd generation), representative samples, after electrophoresis, are displayed on ethidium bromide stained 1% agarose gel. RT-PCR products for T-phylloplanin-GFP with the expected 1304 bp band for independent line numbers T1.1 to T1.4 and T2.1 to T2.2; untransformed tobacco control KY14 (W), positive controls by taking pBS-nat-T-phylloplanin-GFP (P, a clone of the chimeric nat-T-phylloplanin-GFP gene in Bluescript) used as template are shown. (b) Expression analysis (mRNA levels) of independent transgenic tobacco lines (R_1_ progeny, 2nd generation) containing the native T-phylloplanin-GFP construct by real-time quantitative reverse transcriptase-polymerase chain reaction (qRT-PCR) in stably transformed tobacco. Independent plant lines with Kan^R^ : Kan^S^ = 3 : 1 were selected for analysis. The relative expression levels of nat-T-phyllo-GFP-specific mRNA in independent transgenic tobacco lines were analyzed using the comparative threshold cycle (Ct) method and were presented as fold-changes compared with the reference transgenic line #R. Independent tobacco lines (T1.1 to T1.4 and T2.1 to T2.2) are indicated on the histogram. Wild-type plants were used as the reference (R) line. Data are expressed as mean ± S.D. of 5 observations. The amplified RT-PCR products of the nat-T-phylloplanin-GFP gene in each independent line showed similar melting curves with a Tm of 79.9°C, indicating product homogeneity, whereas the internal control tubulin gene had a Tm of 81.7°C (data not shown). (c) Western blot analysis of transgenic lines expressing the chimeric nat-T-phylloplanin-GFP gene probed with GFP-specific polyclonal antibodies showed the expected bands as marked (145 kD). The nat-T-phylloplanin-GFP band was detected with polyclonal GFP-antibodies in the independent transgenic lines (T1.1 to T1.4 and T2.1 to T2.2); no band was detected in wild-type tobacco (*N. tabacum* cv. KY14) plants (W).

The chimeric nat-T-phyllo-GFP gene (Figure 1) was introduced into tobacco (*Nicotiana tabacum* cv. KY14) plants by* Agrobacterium*-mediated transformation. We developed ten independent transgenic lines from each of the two constructs, pKM24-ibm8 and pKM24-ibm10. The independent primary transgenic lines (R_0_ plants) were assayed for gene integration by PCR analysis (data not shown). Reverse transcriptase-PCR (RT-PCR) analysis of transgenic R_1_ and R_2_ progeny gave the expected 1304 bp fragments derived from nat-T-phyllo-GFP, showing the stable integration and expression of the nat-T-phyllo-GFP gene in the transgenic KY 14 tobacco genome (data presented only for R_1_ progeny; Figure 2(a)). Examination of the nat-T-phyllo-GFP protein load in transgenic plants was estimated via Western blot assay against the C-terminal fused GFP using primary anti-GFP antibodies (Figure 2(b)). To determine the degree of nat-T-phyllo-GFP expression in the transgenic plants among the independent lines, we examined transcript abundance by real-time qRT-PCR (Figure 2(c)). As is typically observed, the relative level of the fused synthetic chimeric nat-T-phyllo-GFP-specific mRNA varied by approximately 136- to 458-fold in group-I transgenic plants with tobacco-optimized phylloplanin and by approximately 22- to 121-fold in group-II transgenic lines with grass optimized phylloplanin (Figure 2(b)).

**Figure 3:**
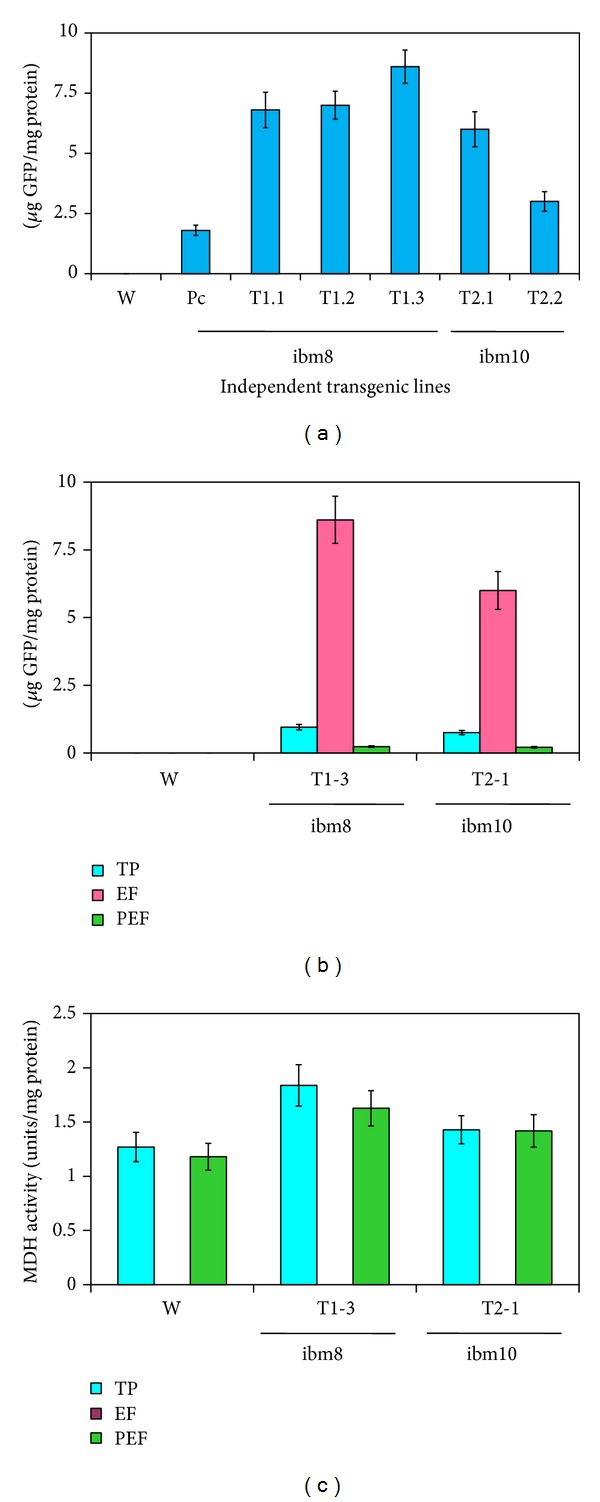
Cell wall targeted analysis of fused nat-T-phylloplanin-GFP in transgenic tobacco (*Nicotiana tabacum* cv. KY 14) lines. (a) The GFP concentration (expressed as *μ*g GFP per mg protein) measured in leaf extracellular fluid (EF) of transgenic and wild-type plants. GFP was measured with the Turner Biosystems Luminometer by using the GFP-UV module. (b) Comparison of GFP concentration (*μ*g GFP per mg protein) in total soluble protein (TP), extracellular fluid (EF), and postextracellular fluid (PEF) fractions measured in leaf samples of transgenic and wild-type plants. GFP was measured with the Turner Biosystems Luminometer by using the GFP-UV module. (c) Malate dehydrogenase (MDH) activity (expressed in units per mg protein) in total protein (TP), extracellular fluid (EF), and postextracellular fluid (PEF) fractions measured in leaf samples of transgenic and wild-type plants. Error bars indicate the standard deviation of readings from five different plants of each line (three readings per plant). W: wild-type KY14; GFP: pKM24KH-GFP transgenic plant expressing only GFP; T-phylloplanin-GFP transgenic lines: ibm8T1.1 to ibm8T1.4 and ibm10T2.1 to ibm10T2.2.

**Figure 4:**
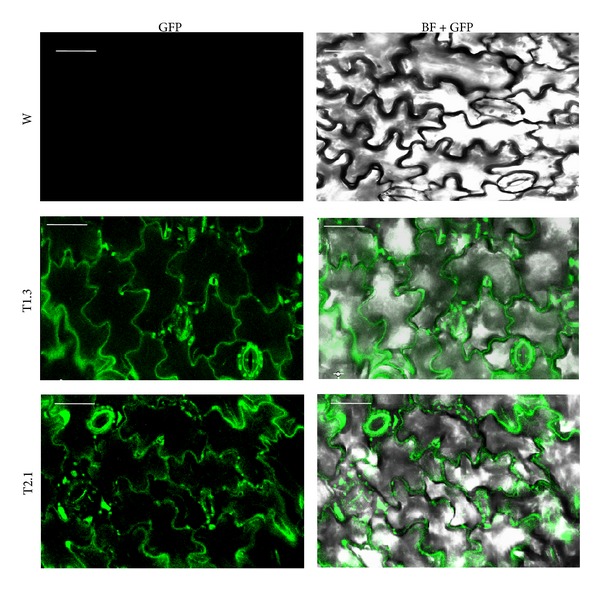
Localization analysis of cell wall targeted nat-T-phyllo-GFP by confocal laser scanning microscopy. Leaves of transgenic native T-phylloplanin-GFP plants expressing GFP (green fluorescence) visualized by confocal laser scanning microscopy (200x magnification). No GFP fluorescence was detected in leaf cells of wild-type (W) plants. GFP and GFP-bright field colocalized images were taken from leaf sections of wild-type (W) and transgenic (T1.3 and T2.1) plants. Scale bar represents 56 *μ*m on all images.

Transgenic lines expressing the fused synthetic chimeric nat-T-phyllo-GFP gene were also evaluated by quantifying GFP fluorescence using a spectrofluorometric assay [30] (Figures 3(a) and 3(b)) and by fluorescent laser confocal microscopy (Figure 4). GFP was quantified in total soluble protein (TP), extracellular fluid (EF), and postextracellular fluid (PEF) fractions from leaves of transgenic plants generated with pKM24-ibm8 and pKM24-ibm10. A higher GFP concentration was found in the EF as compared to the TP and PEF fractions (Figure 3(b)). Malate dehydrogenase (MDH) activity was measured in the TP, EF, and PEF fractions, and there was no detectable MDH activity in the EF fraction (Figure 3(c), Table 2). Confocal microscopy also showed GFP fluorescence in the apoplast region (Figure 4). Independent transgenic lines showing good expression were selected for further analysis. In this study, wild-type untransformed plants, the plants transformed with the empty vectors, and the vector-GFP construct were used as controls. Plants transformed with the empty vector and the vector-GFP construct behaved the same as the untransformed wild-type plants (data not shown).

### 3.2. Overexpression of T-phyllo-GFP Inhibits* P. tabacina *Spore Germination and Leaf Infection

**Figure 5:**
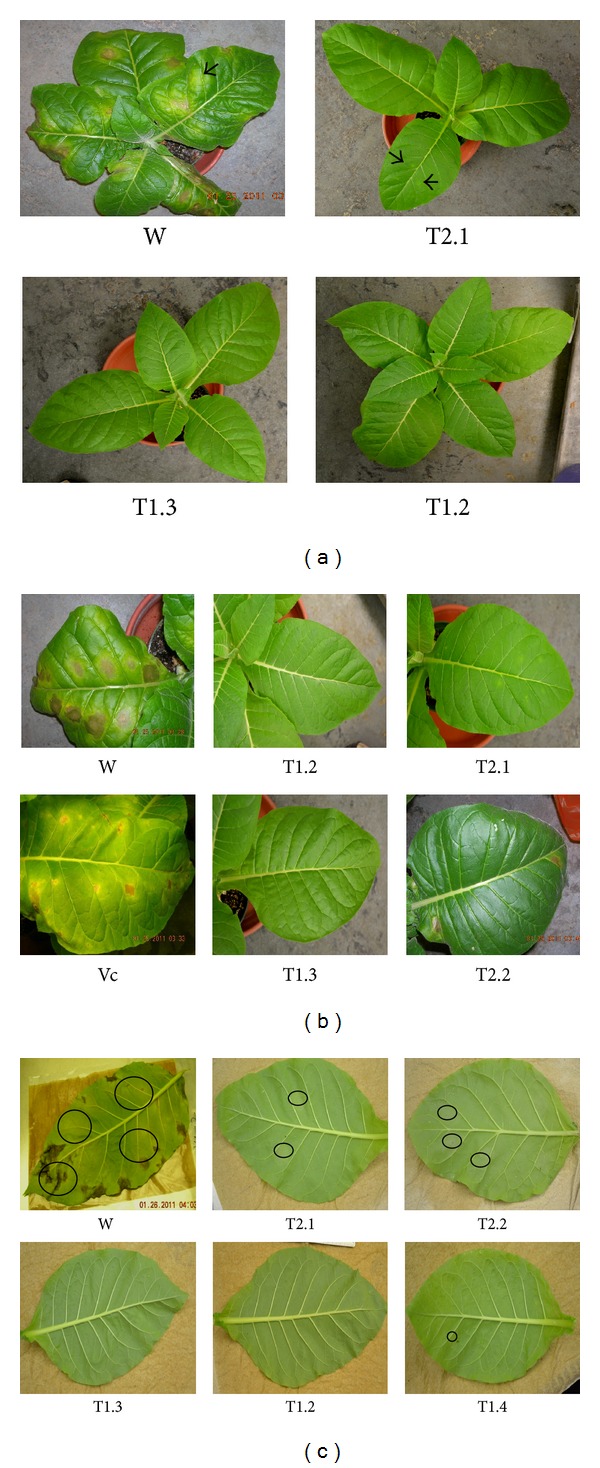
Assay of blue mold infection in transgenic tobacco plants expressing the nat-T-phylloplanin-GFP gene fusion. Response of mature control tobacco plants (*Nicotiana tabacum* cv. KY 14) and three transgenic lines (ibm8T1.2, ibm8T.3, and ibm10T2.1) to* Peronospora tabacina* infection 7 days postinoculation. (a) The untransformed wild-type tobacco cultivar KY14 is highly susceptible to infection. No infection was observed on ibm8T1.2 and ibm8T1.3 plants, and minimal infection was observed on plants of transgenic line ibm10T2.1. (b) Leaves of wild-type KY14 (W) and transgenic (T1.2, T1.3, and T2.1) plants seven days postinoculation with* Peronospora tabacina* sporangia. (c) Sporulation on leaves of wild-type KY14 (W) and transgenic (T1.2, T1.3, and T2.1) lines seven days postinoculation with* P. tabacina*. Leaves were removed from the plants and placed overnight inside plastic tubs to initiate sporulation.

**Figure 6:**
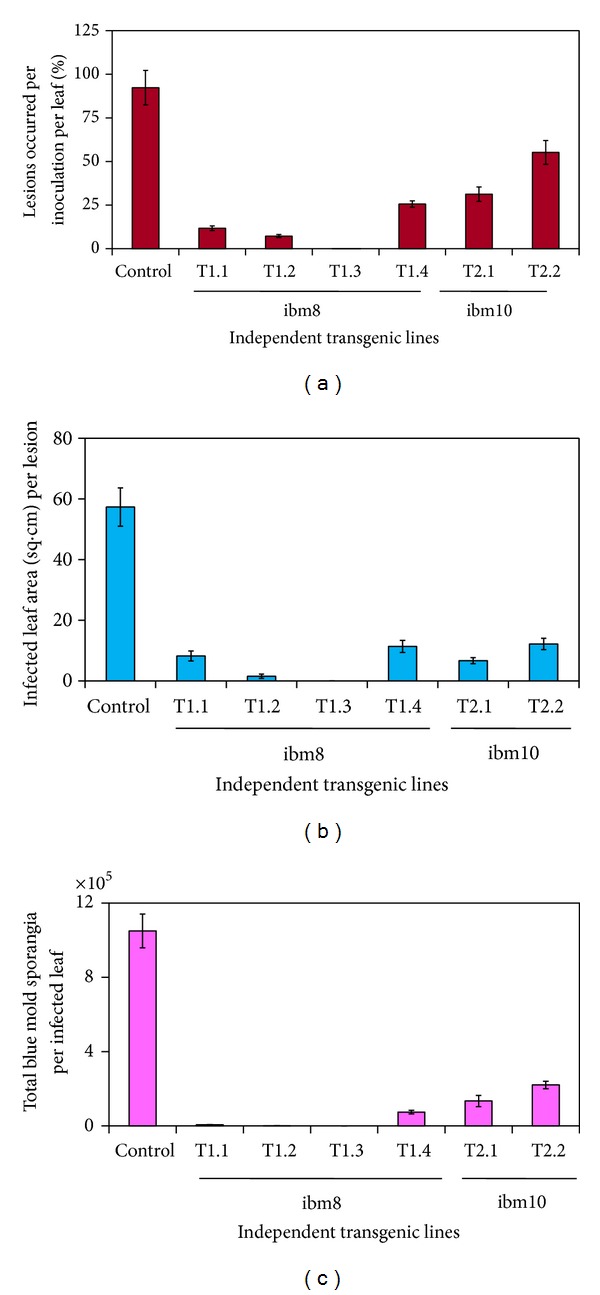
Analysis of blue mold infection in transgenic plants expressing the chimeric nat-T-phylloplanin-GFP fusion. Blue mold sporulation on leaves of the wild-type (W;* Nicotiana tabacum* cv. KY 14) and transgenic (T1.2, T1.3, and T2.1) plant lines 7 days postinoculation with sporangia of* Peronospora tabacina*, following overnight incubation in sealed plastic tubs. (a) Evaluation of sporulation on leaves (percentage of lesions that occurred per inoculation per leaf) in wild-type and transgenic plants. (b) Evaluation of sporulation on leaves (infected leaf area in sq. cm. per lesion) in wild-type and transgenic plants. (c) Evaluation of sporulation on leaves (total blue mold sporangia per infected leaf) in wild-type and transgenic plants.

Due to over expression of nat-T-phyllo-GFP, blue mold infection (as determined by lesion formation) was dramatically decreased from 87–100%, with a marked reduction in spore count of 99-100% and a decrease in the infected area between 86 and 100% in transgenic KY 14 tobacco plants (codon optimized for tobacco) as compared to wild KY14 plants (Figures 5 and 6). However, in transgenic KY 14 tobacco plants expressing the nat-T-phyllo-GFP construct codon optimized for creeping bentgrass, lesion formation was inhibited from 40–66%, with a 79–87% reduction in spore count and a 79–88% decrease in lesion area in comparison to wild-type KY 14 tobacco plants (Figures 5 and 6).

## 4. Discussion

Genes encoding defense proteins have already been employed to boost plant resistance against fungal and bacterial phytopathogens [34, 35]. A significant effort has been directed toward the identification and characterization of antifungal proteins and their expression in transgenic plants [36]. For instance, the expression of the defense-related gene ch5B encoding a chitinase reduced disease symptoms of* Botrytis cinerea* in strawberry [37], transgenic orange plants expressing a tomato thaumatin-like protein exhibited better tolerance toward* Phytophthora citrophthora* [38], constitutive overexpression of an antimicrobial protein gene, Ace-AMP1, from* Allium cepa* in* Oryza sativa* increased resistance against major rice pathogens like* Magnaporthe grisea*,* R. solani*, and* Xanthomonas oryzae* [39], and potato plants expressing the snakin-defensin hybrid protein exhibited no above-ground or tuber symptoms of potato ring rot disease caused by the bacterium* Clavibacter michiganensis* [40]. Also, overexpression of CaAMP1 (*Capsicum annuum* ANTIMICROBIAL PROTEIN1) in* Arabidopsis thaliana* conferred broad-spectrum resistance to the hemibiotrophic bacterial pathogen* Pseudomonas syringae*, the biotrophic oomycete* Hyaloperonospora parasitica*, and the fungal necrotrophic pathogens* Fusarium oxysporum* and* Alternaria brassicicola* [41].

Transgenic tobacco expressing different antimicrobial proteins also exhibit enhanced tolerance against fungal and bacterial phytopathogens. A hybrid of the cysteine-rich antimicrobial proteins snakin-1 (SN1) and defensin-1 (PTH1) expressed in tobacco protects the plants from severe anthracnose symptoms caused by the fungus* C. coccoides* [40]. Another study in transgenic tobacco demonstrated that overexpression of a novel small antimicrobial protein LJAMP1 significantly enhanced the resistance of tobacco against not only the fungal pathogen* A. alternate*, but also against the bacterial pathogen* Ralstonia solanacearum*, with no visible alteration in plant growth and development observed [42]. Studies by Alexander et al. [43] demonstrated that although the pathogenesis-related protein 1a (PR 1a) does not have a measurable effect on diseases caused by tobacco mosaic virus or potato virus Y, it significantly reduces the disease severity caused by infection with the oomycete pathogens* Peronospora tabacina* and* Phytophthora parasitica* var.* nicotianae*.

Recently, it has been reported that the apoplast-directed native mature T-phylloplanin protein fused to GFP confers resistance against blue mold better than it does when targeted to the cytoplasm [21]. Hence, in the present study, we used transgenic tobacco (*Nicotiana tabacum* cv. KY14) plants overexpressing a synthetic native T-phylloplanin-GFP fusion (nat-T-phyllo-GFP) protein (with its own native signal peptide) directed to the apoplast to evaluate the antimicrobial activity of the fusion protein. The coding sequences of the chimeric gene constructs that were codon optimized for either a dicot (tobacco) or a monocot (creeping bentgrass) were placed between the heterologous M24 promoter of the* Mirabilis mosaic virus* [2, 21, 23, 24] and the terminator sequence from the rbcSE9 gene (Figure 1). It has been documented that the* Mirabilis mosaic virus* full-length transcript promoter is constitutive in nature and is 25-fold stronger than the CaMV35S promoter in transgenic tobacco plants [2, 27, 32]. Regenerated plants were screened for kanamycin resistance and then by further molecular analysis [24]. The nat-T-phyllo-GFP-positive, kanamycin-resistant plantlets were obtained and grown in the greenhouse. The transgene insert copy numbers in the independent transformant lines (R_0_) were estimated by examining the segregation of kanamycin resistance in the self-pollinated progeny resulting (R_1_). Ten transgenic lines that showed Mendelian inheritance ratios of 3 : 1 (Kan^R^ : Kan^S^) in the progeny were considered to be carrying a single copy of the transgene. Then the isolation of homozygous transgenic plants with a single insert of the transgene was conducted in the progeny self-fertilized from R_1_ detected by nat-T-phyllo-GFP analysis. We randomly selected six transgenic R_2_ lines that carried a single insert of the transgene for subsequent molecular analyses. Quantitative RT-PCR and Western blot analyses showed different levels of accumulation of nat-T-phylloplanin-specific transcripts and the synthetic nat-T-phylloplanin-GFP fusion protein in transgenic plants (Figure 2). Strong expression of the synthetic nat-T-phyllo-GFP gene was observed in the transgenic ibm8- (1.2 and 1.3) and ibm10-derived (2.1) lines, with moderate to weak expression in ibm8 (1.1 and 1.4) and ibm10 (2.2) lines. Western blot analysis showing the expected 45 kD protein, GFP quantification, and visualization by confocal microscopy indicated that the nat-T-phyllo-GFP construct is expressed stably in transgenic tobacco plants. To evaluate blue mold disease resistance in the transgenic tobacco plants, the standard leaf assay for* P. tabacina* infection was performed. The transgenic lines overexpressing nat-T-phyllo-GFP and the empty-vector or untransformed control plants were challenged with the oomycete pathogen* P. tabacina*. Although the appearance of blue mold symptoms, caused by* P. tabacina*, was observed in some experimental groups, the severity was significantly lower in plants expressing the nat-T-phyllo-GFP gene (Figures 5 and 6). From the disease index, ibm8 (1.2 and 1.3) showed the highest resistance against* P. tabacina*, and ibm8 (1.1 and 1.4) and ibm10 (2.1 and 2.2) also exhibited a significant increase of disease resistance compared with the control (Figures 5 and 6). The disease resistant transgenic ibm8 (1.3) and ibm10 (2.1) lines were further extensively evaluated by confocal microscopy, which again revealed the presence of the chimeric nat-T-phylloplanin-GFP fusion protein in the apoplast region. MDH activity in extracellular fluid (EF) from leaf samples of transgenic ibm 8 (1.3) and ibm10 (2.1) lines was undetectable, which again shows that EF was not contaminated with cytosolic proteins and has a higher concentration of phylloplanin fused to GFP. Our data indicate that transgenic plants display resistance only if they express the antifungal phylloplanin-GFP protein fusion at levels over 6 to 8 *μ*g per mg of extracellular or apoplastic protein.

Overexpression of the chimeric native T-phylloplanin-GFP gene in transgenic tobacco plants resulted in dramatically decreased lesion formation and spore count, as well as a reduction in the size of the infected area, in transgenic KY14 tobacco plant lines generated with both the tobacco-optimized and bentgrass-optimized genes, in comparison to the KY14 control. However, plants of transgenic KY14 line ibm10T2 (codon-optimized for bentgrass) showed less blue mold resistance in comparison with plants of line ibm8 (codon-optimized for tobacco). These results demonstrate the important role that phylloplanin plays in controlling blue mold infection; however, the significance of codon optimization for tobacco cannot be ruled out.* P. tabacina *is an oomycete pathogen that reproduces by airborne vegetative sporangia, and the initial host contact and spore deposition occur at the phylloplane [17, 18]. Our data strongly suggest a plant protective role for phylloplanin protein against pathogen infection, and it can be concluded that expression of phylloplanin increases disease resistance against* P. tabacina*. In a similar study, it has been shown that high-level expression of PR-la in transgenic tobacco results in tolerance to infection by* P. tabacina*, again demonstrating that host plants overexpressing a defense protein have increased tolerance against pathogenic organisms [43].

It has been suggested that tobacco has two surface-disposed mechanisms for inhibiting* P. tabacina *disease: the first is the SGT-produced T-phylloplanins and the second is the abundant diterpenes and T-phylloplanins produced by tall trichomes on older leaves [19]. Microarray transcriptome analysis of different gene expression levels in tobacco leaf trichomes showed a 22-fold enrichment of T-phylloplanin in trichomes [44]. It has been reported that phylloplanins are not unique to tobacco but are also present on the leaf surfaces of other plants. The phylloplanin levels are very high, moderate-to-high, moderate, and low in tobacco, jimson weed, sunflower, and soybean, respectively. However, the relative abilities of leaf water washes (LWWs) from these plants to inhibit* P. tabacina *spore germination and leaf infection were found to be sunflower > tobacco > jimson weed, with no activity from soybean [19].

It has been reported that tobacco (*Nicotiana tabacum* cv. KY14) contains less phylloplanin I to IV than other varieties [17, 18]. Therefore, in the present study we increased the blue mold resistance of KY14 by overexpressing T-phylloplanin. It has been shown that LWWs containing phylloplanin immediately arrest the germination of sporangia and also tube growth and development in* P. tabacina* [17, 18]. Furthermore, studies using GUS/GFP reporter genes and the T-phylloplanin promoter demonstrate that T-phylloplanins are produced locally in SGTs and are secreted onto the leaf surface, where they dissolve in TGST exudate and are dispersed widely on the leaf surface as a result of exudate flow [17, 18]. RNAi-mediated knockdown of the T-phylloplanin gene results in increased plant susceptibility to* P. tabacina *infection [45]. In the present study, using an apoplast-targeting sequence, we could overexpress synthetic nat-T-phyllo-GFP in the apoplast region where it strengthens the host-defense system and inhibits* P. tabacina *spore germination and leaf infection. It is noteworthy that a combination of T-phylloplanins and high TGST exudates may provide maximal inhibition of spore germination as demonstrated by overexpressing the native mature tobacco phylloplanin protein without its signal peptide fused to GFP [21] or the synthetic native phylloplanin with its own native signal peptide fused to GFP (present study). The engineered secretion of candidate defense proteins on leaf surfaces might enhance disease resistance [46], and in the current study the overexpression of synthetic nat-T-phylloplanin in the blue mold-susceptible tobacco variety KY 14 has been shown to increase plant resistance to* P. tabacina*. Such strategies could be a valuable tool in increasing the capacity for the elimination of susceptible individuals and lines during early stages of plant breeding programs.

## Figures and Tables

**Table 1:** DNA sequences of oligonucleotide primers used for RT-PCR and qRT-PCR analysis of the T-phyllo-GFP gene in transgenic plants.

Primer name	Sequence (5′-3′)
5′ T-phyllo-GFP#1	ATG GGT ATA CTT GTT CCA ACA
3′ T-phyllo-GFP#2	TCA CTT GTA CAG CTC GTC CAT
3′ T-phyllo-GFP#3	GAG TTG CAA CCA CTA AAT TGC
5′Tub#4	ATG AGA GAG TGC ATA TCG AT
3′Tub#5	TTC ACT GAA GAA GGT GTT GAA

**Table 2:** MDH activity (units/mg protein) in TP (total protein fraction), EF (extracellular fraction), and PEF (postextracellular fraction) from leaves of the control (KY14), the transgenic tobacco line overexpressing the tobacco-optimized phylloplanin (T1.3), and the transgenic tobacco line overexpressing the grass-optimized phylloplanin (T2.1).

	TP	EF	PEF
Control	1.27 ± 0.134	0.003 ± 0.0002	1.18 ± 0.124
Ibm8 (T1.3)	1.832 ± 0.191	Not detected	1.627 ± 0.163
Ibm10 (T2.1)	1.429 ± 0.13	Not detected	1.418 ± 0.15
